# PTSD and Partial PTSD among First Responders One and Five Years after the Paris Terror Attacks in November 2015

**DOI:** 10.3390/ijerph20054160

**Published:** 2023-02-25

**Authors:** Clémentine Prioux, Maude Marillier, Cécile Vuillermoz, Stéphanie Vandentorren, Gabrielle Rabet, Matthieu Petitclerc, Thierry Baubet, Lise Eilin Stene, Philippe Pirard, Yvon Motreff

**Affiliations:** 1 Santé Publique France, Direction des Maladies non Transmissibles et Traumatismes, F-94415 Saint-Maurice, France; 2 INSERM, Institut Pierre Louis d’Epidémiologie et de Santé Publique (IPLESP), Department of Social Epidemiology, Sorbonne Université, F-75012 Paris, France; 3 INSERM, Bordeaux Population Health Research Center, University of Bordeaux, U1219, F-33000 Bordeaux, France; 4 Santé Publique France, Direction Scientifique et Internationale, F-94415 Saint-Maurice, France; 5 Santé Publique France, Direction Appui, Traitements et Analyses des Données, F-94415 Saint-Maurice, France; 6 Service Médical D’urgence—Bureau de Santé et de Prévention, Brigade de Sapeurs-Pompiers de Paris, 1, Place Jules-Renard, F-75017 Paris, France; 7 Laboratoire UTRPP, Université Sorbonne Paris Nord, F-93430 Villetaneuse, France; 8 APHP Hôpital Avicenne, Psychopathology Department for Children, Adolescents, General Psychiatry and Specialized Addiction, F-93009 Bobigny, France; 9 Centre National de Ressources et de Résilience Lille-Paris (CN2R), F-59000 Lille, France; 10 Norwegian Centre for Violence and Traumatic Stress Studies (NKVTS), NO-0409 Oslo, Norway; 11 CESP, INSERM, MOODS team, Faculté de Médecine Paris–Saclay, Université Paris–Saclay, F-94275 Le Kremlin Bicêtre, France

**Keywords:** posttraumatic stress disorder, trauma, terrorist attacks, first responders, longitudinal data

## Abstract

Following the Paris terror attacks in November 2015, a large number of first responders (FR) were mobilized and consequently were at risk of developing posttraumatic stress disorder (PTSD). Based on the ESPA 13 November survey, the objectives of this study were to 1) describe the prevalence of PTSD and partial PTSD in FR five years after the attacks, 2) describe the changes in PTSD and partial PTSD from one to five years after the attacks, and 3) examine factors associated with PTSD and partial PTSD five years after the attacks. Data were collected using an online questionnaire. PTSD and partial PTSD were measured using the Post-Traumatic Stress Disorder Checklist based on the DSM-5 (PCL-5). Gender, age, responder category, education level, exposure, mental health history, history of traumatic events, training, social support, concern about the COVID-19 epidemic, and somatic problems present after the attacks were all analyzed as potential factors associated with PTSD and partial PTSD using multinomial logistic regression. A total of 428 FR were included 5 years after the attacks, of which 258 had participated also 1 year after the attacks. Five years after the attacks, the prevalence of PTSD and partial PTSD were 8.6% and 22%, respectively. Presence of somatic problems after the attacks were associated with PTSD. Involvement in dangerous crime scenes was associated with a higher risk of partial PTSD. No awareness of psychological risks in the context of professional activity through specific training was associated with partial PTSD, in particular among participants aged 45 years or more. To mitigate PTSD for FR, monitoring mental health symptoms, providing mental health education, and providing treatment may be needed for several years after the attacks.

## 1. Introduction

During the 13 November 2015, attacks that took place in Paris and Saint-Denis, 130 people were killed, hundreds suffered from serious physical injuries, and thousands of people were exposed directly or indirectly to the event [1]. Thousands of first responders (FR) were mobilized on the day of the attacks but also in the days and weeks that followed [2].

The experience of such potentially traumatic events can have an impact on the health and quality of life of people who have been exposed to them [3]. These impacts can manifest as the development of psychological and/or physical disorders [4,5,6] that may persist over time. Posttraumatic stress disorder (PTSD) [7,8], including partial PTSD that occurs in individuals who have developed PTSD symptoms without meeting all of the diagnostic criteria for PTSD [9], is one example.

Most of the scientific literature focuses on the psychological impact on civilians and direct victims affected by an attack. So far, only a few post-attack studies have been conducted in Europe on FR with a measurement point of about one year after the attacks [10]. Indeed, attack-related psychological disorders such as PTSD change over time, and improving knowledge on mental health evolution is needed [11]. In addition, intentional events such as terrorism lead to a greater persistence of PTSD symptoms over time compared to unintentional events [12]. The bulk of studies have shown an increase in PTSD symptoms among FR, such as among FR to the 2001 World Trade Centre (WTC) terrorist attacks, with PTSD prevalence of 12.1% in wave 1 (2–3 years after the attacks) and 19.5% in wave 2 (5–6 years after the attacks). This increase over time is even more pronounced among so-called ‘non-traditional’ FR [13,14].

The evolution of PTSD may depend on the population affected, the nature of the event, and individual factors [15], but studies suggest that variability between individuals can be captured by a few trajectories [11]. In particular, PTSD risk trajectories were studied among WTC FR. For example, 8 years after the World Trade Center attacks, the prevalence of delayed expression of PTSD was 8.5% among police officers and 6.7% among non-traditional WTC FR (construction workers, maintenance/repairs workers, security guards, etc.) [13]. Several risk and protective factors for PTSD among FR have been found in studies conducted 4 and 6 years after the 2001 WTC terrorist attacks. Low social support, event-related job loss, psychiatric history, comorbid depression and anxiety, presence of somatic symptoms and medical problems diagnosed after the attacks, presence of life stressors, having received only basic first aid training, as well as having responded to unsecured crime scenes are known to be risk factors for chronic or delayed onset PTSD [12,13,16,17]. However, data about PTSD trajectories and associated factors to long term PTSD are lacking in Europe. More research is needed across different settings and countries.

The objectives of this study were to (1) describe the prevalence of PTSD and partial PTSD in FR five years after the November 2015 Paris attacks, (2) describe the changes in PTSD and partial PTSD from one to five years after the attacks, and (3) examine factors associated with PTSD and partial PTSD five years after the attacks.

## 2. Materials and Methods

### 2.1. Participants and Recruitment

The ESPA 13 November survey [2,18,19] is an open cohort study of people exposed to the Paris attacks in November 2015. The first wave of the survey was conducted between 8 and 12 months after the attacks, between 7 July 2016 and 10 November 2016, and the second wave was conducted 5 years after the attacks, between 16 November 2020 and 6 April 2021, while the COVID-19 pandemic was going on but before the trial of the attacks, which took place between September 2021 and June 2022.

In order to be able to participate in the first and second phase of the ESPA 13 November survey, participants had to complete an online inclusion questionnaire after providing their consent. Three inclusion criteria were requested: being older than 18 years, having intervened on the night of 13 November and/or during the following 3 weeks in contexts related to the terrorist attacks, and meeting criterion A of the DSM-5 (Diagnostic and Statistical Manual of Mental Disorders, fifth edition) definition of PTSD. Subsequently, the epidemiological survey in the form of an online questionnaire was offered to participants meeting the inclusion criteria. Participants were not required to have completed the first wave of the survey in order to participate in the second. 

The survey was offered to different categories of FR: health professionals, affiliated volunteers from civil protection associations such as the Civil Protection of Paris, the French Red Cross, the Order of Malta, and also the Paris fire brigade, the police, and the staff of the Paris and Saint-Denis town halls. 

First-aid workers or volunteers (excluding firefighters) who had provided social or medical support to people during the 18 November 2015 police intervention in Saint-Denis (directed against terrorists who participated in the 13 November attacks) were also able to participate.

Information to individuals was provided via the partner institutions of the survey holding their own register of professionals involved, a media campaign, posters, leaflets, videos, and announcements relayed on social networks (Facebook, LinkedIn and Twitter). The 664 FR (police officers, firefighters, volunteers from civil protection associations, health professionals) who participated in the first wave of ESPA 13 November survey were emailed to participate to the second wave [2]. 

### 2.2. Data Collection

Data were collected using a secure online questionnaire accessible via the Santé publique France website. 

#### 2.2.1. Dependent Variable

PTSD and partial PTSD were measured using a self-report scale, the Post-Traumatic Stress Disorder Checklist based on the DSM-5 (PCL-5) [20], a scientifically validated and widely used measurement scale. The criteria are as follows:

- Criterion A: Having had a stressful experience. Matching our inclusion criterion, this refers to the November 2015 terrorist attacks during which survey participants responded.
- Criterion B: Intrusion symptoms
- Criterion C: The symptoms of avoidance
- Criterion D: Symptoms of altered cognitions and moods
- Criterion E: Symptoms of altered wakefulness and responsiveness
- Criterion F: Symptoms described by the previous criteria (B to E) that have lasted for more than one month.

Respondents were asked to rate how they experienced each item in the past month on a 5-point scale ranging from 0 to 4 (0 = Not at all, 1 = A little bit, 2 = Moderately, 3 = Quite a bit, 4 = Extremely). 

With the exception of criterion A, each item with a score of 2 or more (“moderately” or more) was considered a symptom of PTSD. According to the DSM-5 definition, a diagnosis of PTSD requires at least 1 item B (questions 1–5), 1 item C (questions 6–7), 2 items D (questions 8–14), and 2 items E (questions 15–20). Several authors have found good utility in applying the DSM-5 rule to predict a PTSD diagnosis [21,22,23]. Cronbach’s alpha for the 20 PCL-5 items in our sample was 0.93, indicating high internal consistency. Partial PTSD was determined as meeting two or three of DSM-5 criteria B, C, D, or E [24]. Thus, the dependent variable was of the form: no PTSD/ partial PTSD/ PTSD.

#### 2.2.2. Independent Variables

In view of the associations found in the literature regarding factors related to PTSD in FR to terrorist attacks [2,12,13,16,17], several data were collected. In addition to sociodemographic characteristics such as age, gender, and level of education of the FR, most of the data were collected in the same way as for wave 1 [2]: category of first responder; experience of difficulties in professional or personal life in 2015; previous responses to other terrorist acts; knowledge of a resource person within or outside their institution who could help them to deal with the risks and psychological consequences of exposure; perceived sense of social isolation; perceived quality of their day-to-day moral, financial, and social support; and history of mental health. 

Exposure to the events was classified into 3 mutually exclusive categories: 1—Response to unsecured crime scenes on the night of 13 November or during the police assault on 18 November; 2—Response to secured or remote crime scenes on the night of 13 November or 18 November; and 3—Response the day after the events of 13 November or 18 November and/or within 3 weeks after.

In wave 2, information about the experience of potentially traumatic events before the attacks was collected using the following questions (yes/no): “Did you personally experience other traumatic moments before the November 2015 attacks where you felt brutally threatened or that your life was in danger? The events: assault, sexual assault, rape, military combat or war zone experience, prison, and childhood sexual contact with an older person, were classified as intentional traumatic events. The events: natural disaster, serious accident, explosion, fire, and life threatening illness, were classified as unintentional. The responses “other” were reclassified into one of the two previous categories. These same data were collected for traumatic events that occurred after the attacks.

For the second wave, preparedness to face potentially traumatic interventions related to terrorist attacks, which is usually not measured in epidemiological studies after terror attacks, was assessed through two questions asked on preparedness before and after the attacks (never/once/several times): awareness of psychological consequences (overwhelmed stress, PTSD, burnout, etc.) and training to provide psychological first aid following potentially traumatic events. Responses were recoded into two categories: yes (once or several times)/no (never) and then into 3 categories: before and after the attacks/before and/or after the attacks/not before or after the attacks.

For the second wave, the number of somatic problems present after the attacks were collected by asking whether they had suffered from (yes/no): headaches, migraines; osteoarticular problems; stomach aches, spastic colic; asthma or other respiratory problems; gastric ulcer or stomach ache; high blood pressure problems; dermatological problems (eczema, psoriasis, hives); problems with unbalanced diabetes; heart problems (heart attack, angina, angina, chest pain); problems with fatigue or exhaustion; concentration problems; sleep problems; tinnitus (ringing in the ears); other. These data were used to create a continuous variable ranging from 0 to 14 co-occurring somatic problems.

As the data were collected during the COVID-19 pandemics, participants’ concerns about the COVID-19 pandemic were also rated on a scale of 0 “Not at all” to 10 “A lot”. This variable was recoded into 3 modalities: 0–3, 3–7, 7–10 [25]. 

### 2.3. Statistical Analyses

In order to describe the evolution of PTSD between the first and second wave, it was classified into 9 possible trajectories:

- Moderate chronicity: partial PTSD in both wave 1 and 2.
- Severe chronicity: PTSD in both wave 1 and 2.
- Delayed onset PTSD: no PTSD in wave 1 and PTSD in wave 2.
- Delayed onset partial PTSD: no PTSD in wave 1 and partial PTSD in wave 2.
- Partial recovery: PTSD in wave 1 and partial PTSD in wave 2.
- Complete recovery: PTSD in wave 1 and no PTSD in wave 2.
- Remission: partial PTSD in wave 1 and no PTSD in wave 2.
- Worsening: partial PTSD in wave 1 and PTSD in wave 2.
- Resistant: no PTSD in wave 1 and in wave 2.

For each trajectory, we checked that, for each person, the sum of the items of the PCL-5 was in accordance with the trajectory found when applying the DSM-5 criteria to the PCL-5 (e.g., for a complete recovery, partial PTSD at wave 1 and no PTSD at wave 2, the sum of the items of the PCL-5 must decrease). In order to evaluate the intensity of the symptoms, we calculated the mean of the total score at the PCL-5 at wave 1 and wave 2 for each trajectory. 

Before identifying factors associated with PTSD and partial PTSD 5 years after the attacks, we compared participants lost to follow-up with those who participated in both waves in order to study if attrition was associated to sociodemographic or mental health factors. For that, the dependent variable “lost to follow-up (yes/no)” was used in univariate and multivariate logistic. Variables associated with the dependent variable in univariate with a *p*-value < 0.10 were included in the multivariate model. 

Then, to identify factors associated with PTSD and partial PTSD 5 years after the attacks, we performed a multinomial logistic regression model yielding estimates of odds ratios (OR) and their 95% Wald confidence interval limits. Multinomial logistic regression allows taking into account dependent variables coded in more than two non-ordinal modalities. Our dependent variable was coded in 3 modalities: PTSD, partial PTSD, and neither PTSD nor partial PTSD, with the latter modality chosen as the reference. The independent variables presented above were entered into a multivariate model. Based on the existing literature, gender, age, first responder category, education level, degree of exposure to attacks, somatic problems present after the attacks, and concern about the COVID-19 epidemic were included in the model [16,26,27,28]. Regarding the other independent variables, in order to keep the model as parsimonious as possible and because they evaluated phenomena that are relatively close, it was chosen to keep only one variable for each of the following dimensions: mental health history, previous traumatic or difficult events, training/awareness, social support. The choice of variables retained was based on the significance of the association in the multivariate model and/or on expert opinion when multiple variables were of interest. 

The analyses were performed using SAS Enterprise Guide version 7.11 software. 

## 3. Results

### 3.1. Participation Rate and Inclusion

**Figure 1:**
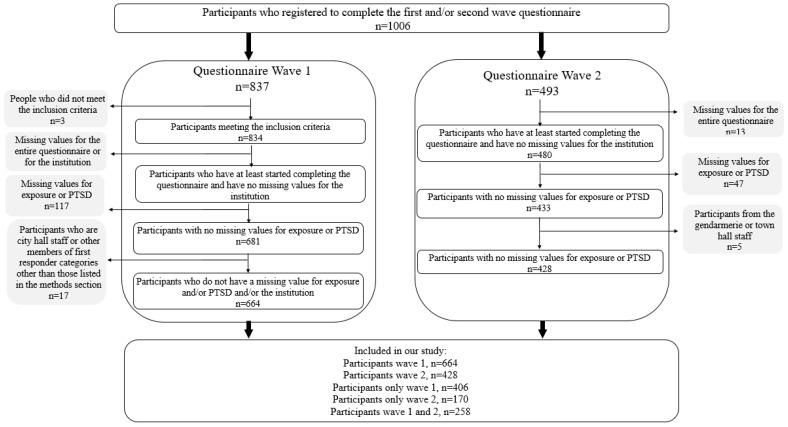
Flowchart, ESPA 13 November survey.

A total of 1330 questionnaires were collected. In total, we obtained 664 participants for wave 1 and 428 participants for wave 2, of whom 406 had only participated in wave 1, 170 in wave 2, and 258 in both waves of the ESPA 13 November survey. The attrition calculated according to our criteria was 61.1%. (Figure 1: Flowchart).

Of the 428 participants in wave 2, 43.9% were firefighters, 25.2% were health professionals, 17.1% were affiliated volunteers and 13.8% were police officers. Women represented 28.7% of the sample and men 71.3%. More specifically, women represented 61.1%, 32.9, 32.2, and 3.3% among health professionals, affiliated volunteers, police forces, and firefighters, respectively. The average age was 41.1 years (standard deviation = 10.2 years; 10 missing values) from 36.5 years old among firefighters to 48.6 years old among health professionals. Sociodemographic characteristics, exposure, mental health history, previous traumatic or difficult events, training, social isolation, and COVID-19 concern by FR category are described in Table S1. Of the 258 participants in both waves, 37.6% were firefighters, 26.4% were health professionals, 19% were affiliated volunteers, and 17.1% were police officers. Women represented 32.6% of the sample and men 67.4%. The average age was 37.9 years (standard deviation = 10.2 years).

### 3.2. Factors Associated to Attrition

In multivariate analysis, attrition was higher among health professionals compared with firefighters (OR = 1.88; 95% CI [1.08–3.26]). It was lower among participants with PTSD compared with those without PTSD (OR = 0.41; 95% CI [0.19–0.88]) and among those reporting low daily social support (OR = 0.53; 95% CI [0.3–0.93]) (Table S2). 

### 3.3. Prevalence of PTSD and Partial PTSD Five Years after the Attacks

The overall prevalence of PTSD 5 years after the attacks in our study sample of FR who participated in the second phase of the ESPA 13 November survey (n = 428) was 8.6%, ranging from 5.5% among affiliated volunteers to 11.9% among police officers. That of partial PTSD was, in total, 22%, ranging from 14.8% among health professionals to 33.9% among police officers (Table 1).

### 3.4. Evolution of PTSD between the 2 Waves

Among FR who participated in wave 1 and wave 2 of the ESPA 13 November survey (n = 258):

- at wave 1, 75.2% of participants did not have PTSD, 17.8% had partial PTSD and 7.0% had PTSD;
- at wave 2, 70.2% of participants had no PTSD, 22.1% had partial PTSD and 7.7% had PTSD.

**Figure 2:**
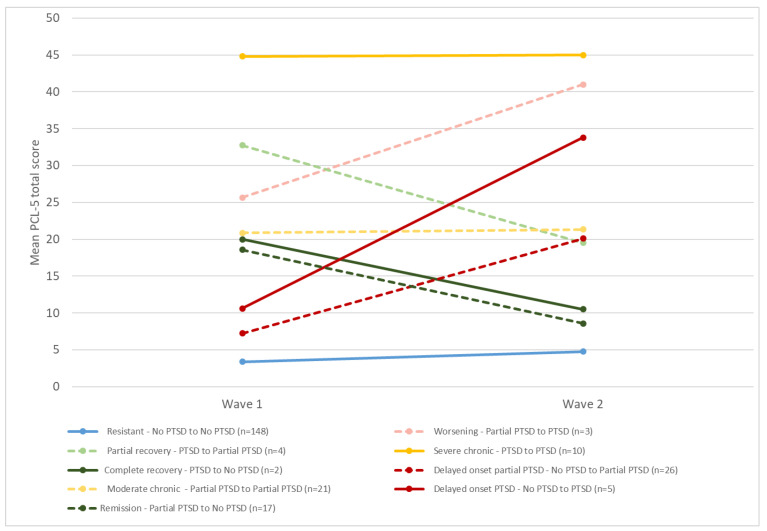
Mean PCL-5 total score in wave 1 and wave 2 according to PTSD category based on DMS-5 criteria, ESPA 13 November survey (n = 238).

The trajectories of PTSD based on DMS-5 diagnostic criteria between waves 1 and 2 are presented in Table 2, and Figure 2 shows the means of the PCL-5 total score in wave 1 and wave 2 according to each trajectory. According to the DSM-5 criteria, one person had partial PTSD at wave 1 and PTSD in wave 2, but had a higher PCL-5 total score in wave 1 (32 versus 21). We found a similar result for another person who had no PTSD in wave 1 and partial PTSD in wave 2: the PCL-5 total score in wave 1 was higher (20 versus 13). For the others with trajectories showing a change in PTSD status between wave 1 and wave 2 according to DSM-5 criteria, there were no discrepancies in the PCL-5 total score for each FR. Sixty-one percent of our sample had a resistant trajectory, 8.9% a moderate chronic trajectory, 4.3% a severe chronic trajectory, 11.6% a delayed onset partial PTSD trajectory and 2.3% a delayed onset PTSD trajectory.

### 3.5. Factors Associated with PTSD and Partial PTSD Five Years after the Attacks 

In multivariate analysis (Table 3), gender, education level, responder category, and history of antidepressant use were not associated with either PTSD or partial PTSD. The association between PTSD or partial PTSD and social isolation adjusted for other factors was borderline significant.

Exposure to unsecured crime scenes on the night of 13 November 2015 was associated with partial PTSD (OR= 3.56; 95% CI [1.55–8.16]) and was borderline significant for PTSD. Experience of an intentional traumatic event prior to the attacks was associated with partial PTSD (OR= 2.4; 95% CI [1.05–5.47]).

The effect of awareness of psychological risks in the context of professional activity through specific training on the probability of developing PTSD or partial PTSD differed according to the age of the responder. The effect is significant for older ages (45 years or older) but not significant for younger ages (under 45 years). For example, among 45-year-old participants, compared with pre- and post-attack awareness, no pre- and post-attack awareness was associated with partial PTSD (OR= 4.77; 95% CI [1.92–11.86]).

On the date of wave 2, high concern for the COVID-19 outbreak (7–10) was associated with PTSD (OR= 6.4; 95% CI [1.7–24.8]), compared with low concern (0–3). 

For an increase in the number of health problems other than psychological after the attacks, the risk of PTSD increased among participants (OR= 2.01; 95% CI [1.55–2.61]).

## 4. Discussion

### 4.1. Main Findings

The present study is the second longitudinal study conducted in France among FR following terrorist attacks [29] and the first to have a measurement point at 5 years after the attacks. 

Analysis of factors of attrition indicated that long-term participation was higher in participants with PTSD compared to those without PTSD. This result suggests a probable selection bias with an ensuing overestimation of PTSD in wave 2 compared to wave 1. To our knowledge, only few studies have addressed the non-response bias among people exposed to terrorist attacks and including FR [30]. A study investigating the importance of high response rates in PTSD research among employees exposed to an industrial disaster (explosion/fire) found that resistance, i.e., the degree of refusal to undergo the primary examination, was strongly related to the severity of the outcome (PTSD) at 7 months. Furthermore, in many individuals who developed PTSD in later waves, initial resistance to participating in the first wave was related to psychological defense mechanisms such as re-experience avoidance in acute post-traumatic stress disorder [31], thus suggesting a likely underestimation of PTSD in wave 1. However, on the other hand, it is possible that individuals who were less exposed to the attacks were less likely to participate because they felt less legitimate to participate. We also found in the multivariate analysis that attrition was lower among participants reporting low perceived daily social support. In studies of terrorist attacks among civilians, the opposite association has been found [30,32]. Attrition was higher among health professionals compared to firefighters, which can be explained because firefighters benefited from a greater awareness campaign in the second wave of the ESPA 13 November survey compared to other categories of FR. In addition, the second wave of the ESPA 13 November survey was conducted during the COVID-19 epidemic. Health professionals were heavily mobilized during this period, which may have had an impact on their participation in the survey.

Firefighters are heavily represented in our sample and are the FR category most exposed to unsecured crime scenes. We highlighted that FR most exposed to horror during the attacks were more likely to develop partial PTSD (and borderline significance for PTSD). Thus, it is likely that there is an overestimation of PTSD and partial PTSD in our sample.

Keeping in mind the previous remarks on factors associated with attrition, our results show that the prevalence of PTSD and partial PTSD seemed to have increased from one year to five years after the attacks among all categories of FR. The prevalence of PTSD in our sample increased from 4.8% in wave 1 [2] to 8.6% in wave 2, and the prevalence of partial PTSD increased from 15.7 to 22% in wave 2. Although the difference between first responder categories was not statistically significant, we observed that the highest prevalence of PTSD 5 years after was among police officers (11.9%), followed by firefighters (9.6%), which doubled between the two waves [2]. The prevalence of partial PTSD increased most among police officers and affiliated volunteers, reaching 33.9 and 27.4%, respectively. Our results are similar to the results of the first wave and are consistent with the IMPACTS study mentioned above, where police officers and French Red Cross volunteer rescuers are the most affected by the psychotraumatic impact of the January 2015 attacks in Paris [29,33]. They are also consistent with a recent review of the literature on PTSD after terrorist attacks, which found that, among FR, the prevalence of PTSD is lowest in the first three years and increases slowly to 10%, reaching a peak approximately 5–6 years after the attack [12]. A study conducted among firefighters 1 to 6 months and then 3 to 4 years after the 2001 WTC terrorist attacks showed that 8.6% of the firefighters had probable PTSD at baseline and 11.1% at follow-up [34]. Another study conducted among police officers found a prevalence of PTSD that was 7.8% 2 to 3 years after the 2001 WTC terrorist attacks and 16.5% 5 to 6 years after [35]. Another study looked at FR and volunteers, lower Manhattan residents, lower Manhattan office workers, and bystanders [17] and found a PTSD prevalence of 12.1% 2 to 3 years after the 2001 WTC terrorist attacks and 19.5% 5 to 6 years after. In their study, rescue workers and volunteers had the following prevalence: 10.8% had delayed expression of PTSD, 8.7% had PTSD chronicization, 3.5% recovered from PTSD between the two waves, and 77.1% did not have PTSD in any waves. The prevalence of chronicization and recovery were similar to the prevalence of the trajectories we had highlighted in our study. In addition, they showed higher prevalence of delayed expression and absence of PTSD in both waves than in our study. In general, the prevalence in the studies of the 2001 WTC terrorist attacks among FR were higher than those we found. However, it may be difficult to compare prevalence rates given the different context and heterogeneous level of exposure of the populations. 

Regarding factors associated with PTSD, the recent literature review cited above highlighted that FR who received only basic rescue training as opposed to intermediate or advanced training would be at greater risk for developing PTSD [12]. In our study, awareness of the psychological consequences that may be experienced after this type of traumatic intervention was significantly associated with PTSD and partial PTSD in the multivariate model for those with advanced age. This reinforces the importance of training and awareness for this type of traumatic intervention on the development of PTSD [2].

Intervention on unsecured crime scenes was found to be a risk factor for partial PTSD and was borderline significant for PTSD. Numerous epidemiological studies after the attacks have also highlighted that the more people are exposed to the horror of a terrorist attack, the more likely they are to develop PTSD, even several years after. This may be explained by the fact that the most exposed FR are the first witnesses of horror and are at the greatest risk of death [12].

We found an association between being highly distressed by the COVID-19 outbreak and PTSD 5 years after the attacks. This association has never been studied in the literature, but longitudinal post-attack studies have found an association among FR between the presence of life stressors and the risk of developing PTSD and partial PTSD [16] as well as PTSD chronicization [13,36,37]. The COVID-19 outbreak may be part of the life stressors that may have been experienced since the outbreak began.

We observed that FR who experienced an intentional traumatic event prior to the attacks were more likely to develop partial PTSD. Some post-attack longitudinal studies of FR have observed this same association for PTSD chronicization [12,37].

Our study highlighted an association between the increase in somatic problems other than psychological after the attacks and PTSD. Post-attack studies of FR have highlighted somatic symptoms and medical problems diagnosed after the attacks as risk factors for PTSD [12,16]. Numerous studies found high comorbidity of PTSD and medical problems that were related to PTSD [13,17]. In addition, patients with PTSD often present with, for example, headaches, sleep disturbances, and pain [38]. Therefore, it is best to be vigilant about the sense of this association, as these somatic symptoms/pathologies can be risk factors for PTSD but also their consequences. However, it may be advisable for healthcare professionals to screen for PTSD when a patient consults for other health problems and if this patient has been exposed to a potentially traumatic event.

The majority of longitudinal or cross-sectional post-attack epidemiological studies with a time point 4–6 years after the events have highlighted social isolation as a risk factor and social support as a protective factor for PTSD [12]. In our study, the association between PTSD or partial PTSD and social isolation adjusted for other factors was borderline significant. We assume that this must be due to a lack of power in our study.

### 4.2. Strengths and Limitations

Some potential biases can be cited [2], such as the healthy worker effect [39], as some of the information about the study was based on hierarchy, colleagues, and occupational medicine. The information and contact protocol on the ESPA 13 November survey was different among responder’s categories. It is possible that information about the survey did not reach certain FR, such as those on sick leave or those who left the institution between the attacks and wave 2 of the survey. Nevertheless, the media campaign and social media posts may have reached some of these FR. It is also possible that FR who were less exposed during the attacks felt they were less legitimate to participate in the survey. 

As with wave 1, given potential recruitment and selection bias, it is not possible to extrapolate our results to the entire responder population. In addition, those lost to follow-up have statistically significant differences from wave 2 participants.

A selection bias may exist due to the use of an online questionnaire: people without internet or not at their ease with such a tool may not participate. However, this tool also brings several advantages [2], such as a reduction of social desirability bias [40], and we assume that our study population that is composed principally of young or middle-aged adults and quite educated is at its ease with such a tool.

Because of the time between the attacks and the second phase of the survey for new participants in the second wave (5 years after the attacks), a likely recall bias may have influenced their reporting for some questions.

Proxy measures (prior use of mental health services) were used to assess mental health history. Mental health disorders are therefore only reflected by those who have sought treatment, and it has been shown that a considerable proportion of people with a psychiatric disorder never present for treatment, and thus can never be diagnosed [41]. 

This study also has some strengths that are important to mention. Firstly, our results document the longitudinal evolution of PTSD in FR after a terrorist attack, largely unexplored in the literature on terrorist attacks in France and more broadly in Europe. Secondly, this study was conducted as an open cohort, allowing the inclusion of new participants 5 years after the attacks. Thirdly, we used a standardized questionnaire (the PCL-5 scale) and questions asked were similar to those used in two other studies in France (the IMPACTS study [29] after the Paris terrorist attacks in January 2015, and the Echos study in Nice [42] after the Nice attack in July 2016) but also in the wave 1 questionnaire, which allowed comparisons.

## 5. Conclusions

Our results confirm that after a terrorist attack, PTSD in FR may persist or emerge within five years after the terrorist attacks. Our study highlights the need for surveillance, assessment, and treatment for FR over many years to prevent and mitigate health consequences. Systematic education and training about awareness of psychological risks in the context of professional activity should be developed. We found evidence among FR that the presence of non-psychological conditions after the attacks was associated with PTSD five years after exposure. This suggests that health professionals such as general practitioners should consider to screen for PTSD when a patient who has been exposed to a terrorist attacks consults for health problems other than PTSD. Due to our small sample, we were unable to determine risk and protective factors for the development of PTSD between the two waves. Further research is therefore needed to investigate longitudinal PTSD trajectories and associated factors, and to evaluate the effectiveness of targeting potentially modifiable risk and protective factors to prevent or mitigate PTSD symptoms. 

## Figures and Tables

**Table 1:** Prevalence of PTSD and partial PTSD by first responder category in wave 2, n = 428.

	PTSD			Partial PTSD		
	n	%	95% CI	n	%	95% CI
Firefighters	18	9.6	5.4–13.8	38	20.2	14.6–25.9
Healthcare professionals	8	7.4	2.4–12.3	16	14.8	8.1–21.5
Affiliated volunteers	4	5.5	0.3–10.7	20	27.4	17.2–37.6
Police officers	7	11.9	3.6–20.1	20	33.9	21.8–46.0
Total	37	8.6	6.0–11.3	94	22.0	18.0–25.9

**Table 2:** Prevalence of trajectories of PTSD between wave 1 and wave 2, n=258.

Trajectory	n	%
Resistant (No PTSD → No PTSD)	158	61.2
Complete recovery (PTSD → No PTSD)	3	-
Remission (Partial PTSD → No PTSD)	20	7.7
Partial recovery (PTSD → Partial PTSD)	4	-
Delayed onset PTSD (No PTSD → PTSD)	6	2.3
Delayed onset partial PTSD (No PTSD → Partial PTSD)	30	11.6
Worsening (Partial PTSD → PTSD)	3	-
Moderate chronic (Partial PTSD → Partial PTSD)	23	8.9
Severe chronic (PTSD → PTSD)	11	4.3
Total	258	100.0

**Table 3:** Multivariate analysis of factors associated with PTSD and partial PTSD among wave 2 FR, n = 342.

	PTSD		Partial PTSD	
	OR	95% CI	OR	95% CI
Gender				
Male	1.00	-	1.00	-
Female	0.75	0.21–2.68	1.15	0.53–2.48
First responder category				
Firefighters	1.00	-	1.00	-
Affiliated volunteers	0.27	0.05–1.38	1.75	0.73–4.16
Police officers	2.19	0.44–10.79	2.44	0.88–6.70
Health professionals	1.24	0.24–6.46	1.60	0.53–4.83
Intervention category (exposure)				
1- The evening of 13 November and at unsecured crime scenes *	3.59	0.99–13.02	3.56	1.55–8.16
2- On the evening of 13 November and at secure or remote crime scenes **	1.00	-	1.00	-
3- Only during the 3 weeks following the attacks	0.42	0.07–2.36	0.99	0.38–2.58
Educational level				
Graduate or post-graduate degree	1.00	-	1.00	-
High school diploma	0.62	0.18–2.06	1.10	0.48–2.49
No high school diploma	0.19	0.03–1.15	0.74	0.26–2.14
Social isolation				
No	1.00	-	1.00	-
Yes	3.14	0.93–10.6	2.27	0.93–5.53
Traumatic events prior to the 2015 attacks				
No	1.00	-	1.00	-
Yes and intentional	1.05	0.25–4.42	2.40	1.05–5.47
Yes and not intentional	1.16	0.38–3.55	1.85	0.93–3.66
History of anti-depressant consumption				
No	1.00	-	1.00	-
Yes	1.33	0.15–11.58	0.69	0.19–2.51
Raising awareness of psychological risks in the context of professional activity through specific training				
25 years				
Before and after the attacks	1.00	-	1.00	-
Before or after the attacks	0.83	0.13–5.46	0.66	0.18–2.41
Neither before nor after the attacks	0.48	0.03–6.75	0.23	0.04–1.28
35 years				
Before and after the attacks	1.00	-	1.00	-
Before or after the attacks	1.97	0.65–5.97	1.26	0.58–2.77
Neither before nor after the attacks	1.41	0.31–6.45	1.05	0.37–2.96
45 years				
Before and after the attacks	1.00	-	1.00	-
Before or after the attacks	4.68	1.11–19.69	2.43	1.12–5.27
Neither before nor after the attacks	4.17	0.57–30.38	4.77	1.92–11.86
Concern about the COVID-19 epidemic (scale of 0 to 10)				
0–3	1.00	-	1.00	-
3–7	1.67	0.51–5.48	1.06	0.55–2.07
7–10	6.40	1.66–24.76	1.52	0.62–3.70
Number of somatic problems present after the attacks of 13 November 2015	2.01	1.55–2.61	1.18	0.99–1.39

* for people who both intervened in unsecured crime scenes (1) and intervened during the 3 following weeks (3), exposure category (1) is retained; ** for people who both intervened in secured crime scenes or were distant from the crime scene during the night of the attack (2) and intervened during the 3 following weeks (3), exposure category (2) is retained.

## Data Availability

The data are not publicly available.

## Supplementary Materials

The following supporting information can be downloaded at: https://www.mdpi.com/article/10.3390/ijerph20054160/s1, Table S1: Sociodemographic characteristics, exposure, mental health history, previous traumatic or difficult events, training and social isolation, COVID-19 concern by first responder category (wave 2 ESPA 13 November survey), n = 428. Table S2: Multivariate regression analysis of the comparison between those who participated only in wave 1 and those who participated in both waves

## References

[1] Philippe J.-M., Brahic O., Carli P., Tourtier J.-P., Riou B., Vallet B. (2016). French Ministry of Health’s Response to Paris Attacks of 13 November 2015. Crit. Care Lond. Engl..

[2] Motreff Y., Baubet T., Pirard P., Rabet G., Petitclerc M., Stene L.E., Vuillermoz C., Chauvin P., Vandentorren S. (2020). Factors Associated with PTSD and Partial PTSD among First Responders Following the Paris Terror Attacks in November 2015. J. Psychiatr. Res..

[3] Bonanno G.A., Brewin C.R., Kaniasty K., Greca A.M.L. (2010). Weighing the Costs of Disaster: Consequences, Risks, and Resilience in Individuals, Families, and Communities. Psychol. Sci. Public Interest.

[4] Bonde J.P.E., Jensen J.H., Smid G.E., Flachs E.M., Elklit A., Mors O., Videbech P. (2022). Time Course of Symptoms in Posttraumatic Stress Disorder with Delayed Expression: A Systematic Review. Acta Psychiatr. Scand..

[5] Hobfoll S.E., Palmieri P.A., Johnson R.J., Canetti-Nisim D., Hall B.J., Galea S. (2009). Trajectories of Resilience, Resistance, and Distress During Ongoing Terrorism: The Case of Jews and Arabs in Israel. J. Consult. Clin. Psychol..

[6] Wesemann U., Bühler A., Mahnke M., Polk S., Willmund G. (2020). Longitudinal Mental Health Effects of the 2016 Terrorist Attack in Berlin on Various Occupational Groups of Emergency Service Personnel. Health Secur..

[7] Kessler R.C. (2000). Posttraumatic Stress Disorder: The Burden to the Individual and to Society. J. Clin. Psychiatry.

[8] van der Velden P.G., Wong A., Boshuizen H.C., Grievink L. (2013). Persistent Mental Health Disturbances during the 10 Years after a Disaster: Four-Wave Longitudinal Comparative Study. Psychiatry Clin. Neurosci..

[9] Berger W., Figueira I., Maurat A.M., Bucassio E.P., Vieira I., Jardim S.R., Coutinho E.S.F., Mari J.J., Mendlowicz M.V. (2007). Partial and Full PTSD in Brazilian Ambulance Workers: Prevalence and Impact on Health and on Quality of Life. J. Trauma. Stress.

[10] Skogstad L., Heir T., Hauff E., Ekeberg Ø. (2016). Post-Traumatic Stress among Rescue Workers after Terror Attacks in Norway. Occup. Med. Oxf. Engl..

[11] Norris F.H., Tracy M., Galea S. (2009). Looking for Resilience: Understanding the Longitudinal Trajectories of Responses to Stress. Soc. Sci. Med..

[12] Rigutto C., Sapara A.O., Agyapong V.I.O. (2021). Anxiety, Depression and Posttraumatic Stress Disorder after Terrorist Attacks: A General Review of the Literature. Behav. Sci..

[13] Pietrzak R.H., Feder A., Singh R., Schechter C.B., Bromet E.J., Katz C.L., Reissman D.B., Ozbay F., Sharma V., Crane M. (2014). Trajectories of PTSD Risk and Resilience in World Trade Center Responders: An 8-Year Prospective Cohort Study. Psychol. Med..

[14] Debchoudhury I., Welch A.E., Fairclough M.A., Cone J.E., Brackbill R.M., Stellman S.D., Farfel M.R. (2011). Comparison of Health Outcomes among Affiliated and Lay Disaster Volunteers Enrolled in the World Trade Center Health Registry. Prev. Med..

[15] Santiago P.N., Ursano R.J., Gray C.L., Pynoos R.S., Spiegel D., Lewis-Fernandez R., Friedman M.J., Fullerton C.S. (2013). A Systematic Review of PTSD Prevalence and Trajectories in DSM-5 Defined Trauma Exposed Populations: Intentional and Non-Intentional Traumatic Events. PLoS ONE.

[16] Pietrzak R.H., Schechter C.B., Bromet E.J., Katz C.L., Reissman D.B., Ozbay F., Sharma V., Crane M., Harrison D., Herbert R. (2012). The Burden of Full and Subsyndromal Posttraumatic Stress Disorder among Police Involved in the World Trade Center Rescue and Recovery Effort. J. Psychiatr. Res..

[17] Brackbill R.M., Hadler J.L., DiGrande L., Ekenga C.C., Farfel M.R., Friedman S., Perlman S.E., Stellman S.D., Walker D.J., Wu D. (2009). Asthma and Posttraumatic Stress Symptoms 5 to 6 Years Following Exposure to the World Trade Center Terrorist Attack. JAMA.

[18] Motreff Y., Pirard P., Vuillermoz C., Rabet G., Petitclerc M., Stene L.E., Baubet T., Chauvin P., Vandentorren S. (2022). Mental Health Care Utilization by First Responders after Paris Attacks. Occup. Med. Oxf. Engl..

[19] Pirard P., Baubet T., Motreff Y., Rabet G., Marillier M., Vandentorren S., Vuillermoz C., Stene L.E., Messiah A. (2020). Use of Mental Health Supports by Civilians Exposed to the November 2015 Terrorist Attacks in Paris. BMC Health Serv. Res..

[20] Weathers F.W., Litz B.T., Keane T.M., Palmieri P.A., Marx B.P., Schnurr P.P. (2013). The PTSD Checklist for DSM-5 (PCL-5). https://www.ptsd.va.gov/.

[21] Ashbaugh A.R., Houle-Johnson S., Herbert C., El-Hage W., Brunet A. (2016). Psychometric Validation of the English and French Versions of the Posttraumatic Stress Disorder Checklist for DSM-5 (PCL-5). PLoS ONE.

[22] Bovin M.J., Marx B.P., Weathers F.W., Gallagher M.W., Rodriguez P., Schnurr P.P., Keane T.M. (2016). Psychometric Properties of the PTSD Checklist for Diagnostic and Statistical Manual of Mental Disorders–Fifth Edition (PCL-5) in Veterans. Psychol. Assess..

[23] Krüger-Gottschalk A., Knaevelsrud C., Rau H., Dyer A., Schäfer I., Schellong J., Ehring T. (2017). The German Version of the Posttraumatic Stress Disorder Checklist for DSM-5 (PCL-5): Psychometric Properties and Diagnostic Utility. BMC Psychiatry.

[24] McLaughlin K.A., Koenen K.C., Friedman M.J., Ruscio A.M., Karam E.G., Shahly V., Stein D.J., Hill E.D., Petukhova M., Alonso J. (2015). Subthreshold Posttraumatic Stress Disorder in the World Health Organization World Mental Health Surveys. Biol. Psychiatry.

[25] Empereur-bissonnet P., Perrine A., Pédrono G., El Haddad M., Zeghnoun A., Richard J., Blanchard M., Saoudi A., Motreff Y., Morel P. (2021). Santé Post Incendie 76—Une Étude à l’écoute de Votre Santé. Étude Épidémiologique Par Questionnaire Sur l’incendie Industriel Du 26 Septembre 2019 à Rouen (France).

[26] Bowler R.M., Han H., Gocheva V., Nakagawa S., Alper H., DiGrande L., Cone J.E. (2010). Gender Differences in Probable Posttraumatic Stress Disorder among Police Responders to the 2001 World Trade Center Terrorist Attack. Am. J. Ind. Med..

[27] Perrin M.A., DiGrande L., Wheeler K., Thorpe L., Farfel M., Brackbill R. (2007). Differences in PTSD Prevalence and Associated Risk Factors among World Trade Center Disaster Rescue and Recovery Workers. Am. J. Psychiatry.

[28] De Stefano C., Orri M., Agostinucci J.M., Zouaghi H., Lapostolle F., Baubet T., Adnet F. (2018). Early Psychological Impact of Paris Terrorist Attacks on Healthcare Emergency Staff: A Cross-Sectional Study. Depress. Anxiety.

[29] Vandentorren S., Pirard P., Sanna A., Aubert L., Motreff Y., Dantchev N., Lesieur S., Chauvin P., Baubet T. (2018). Healthcare Provision and the Psychological, Somatic and Social Impact on People Involved in the Terror Attacks in January 2015 in Paris: Cohort Study. Br. J. Psychiatry.

[30] Vuillermoz C., Stene L.E., Aubert L., Motreff Y., Pirard P., Baubet T., Lesieur S., Chauvin P., Vandentorren S. (2020). Non-Participation and Attrition in a Longitudinal Study of Civilians Exposed to the January 2015 Terrorist Attacks in Paris, France. BMC Med. Res. Methodol..

[31] Weisæth L. (1989). Importance of High Response Rates in Traumatic Stress Research. Acta Psychiatr. Scand..

[32] Stene L.E., Dyb G. (2016). Research Participation after Terrorism: An Open Cohort Study of Survivors and Parents after the 2011 Utøya Attack in Norway. BMC Res. Notes.

[33] Meudal J., Vandentorren S., Simeoni L., Denis C. (2020). French Red Cross Volunteer Rescue Workers: Psychological Characteristics and Healthcare Support after the January 2015 Terrorist Attacks in Paris. J. Nerv. Ment. Dis..

[34] Berninger A., Webber M.P., Niles J.K., Gustave J., Lee R., Cohen H.W., Kelly K., Corrigan M., Prezant D.J. (2010). Longitudinal Study of Probable Post-Traumatic Stress Disorder in Firefighters Exposed to the World Trade Center Disaster. Am. J. Ind. Med..

[35] Bowler R.M., Harris M., Li J., Gocheva V., Stellman S.D., Wilson K., Alper H., Schwarzer R., Cone J.E. (2012). Longitudinal Mental Health Impact among Police Responders to the 9/11 Terrorist Attack. Am. J. Ind. Med..

[36] Feder A., Mota N., Salim R., Rodriguez J., Singh R., Schaffer J., Schechter C.B., Cancelmo L.M., Bromet E.J., Katz C.L. (2016). Risk, Coping and PTSD Symptom Trajectories in World Trade Center Responders. J. Psychiatr. Res..

[37] Maslow C.B., Caramanica K., Welch A.E., Stellman S.D., Brackbill R.M., Farfel M.R. (2015). Trajectories of Scores on a Screening Instrument for PTSD among World Trade Center Rescue, Recovery, and Clean-Up Workers. J. Trauma. Stress.

[38] Sareen J. (2014). Posttraumatic Stress Disorder in Adults: Impact, Comorbidity, Risk Factors, and Treatment. Can. J. Psychiatry Rev. Can. Psychiatr..

[39] Pearce N., Checkoway H., Kriebel D. (2007). Bias in Occupational Epidemiology Studies. Occup. Environ. Med..

[40] Schlenger W.E., Silver R.C. (2006). Web-Based Methods in Terrorism and Disaster Research. J. Trauma. Stress.

[41] Wang P.S., Lane M., Olfson M., Pincus H.A., Wells K.B., Kessler R.C. (2005). Twelve-Month Use of Mental Health Services in the United States: Results from the National Comorbidity Survey Replication. Arch. Gen. Psychiatry.

[42] Bentz L., Vandentorren S., Fabre R., Bride J., Pirard P., Doulet N., Baubet T., Motreff Y., Pradier C. (2021). Mental Health Impact among Hospital Staff in the Aftermath of the Nice 2016 Terror Attack: The ECHOS de Nice Study. BMC Public Health.

