# Supplementary material for: PTSD and Partial PTSD among First Responders One and Five Years after the Paris Terror Attacks in November 2015
Source: Int J Environ Res Public Health. 2023 Feb 25;20(5):4160. doi: 10.3390/ijerph20054160 (PMC10001642; doi:10.3390/ijerph20054160)
Supplement: Supplementary file 1 [file ijerph-20-04160-s001.zip › ijerph-2219965-supplementary.pdf]

Supplementary Materials

Table S1: Sociodemographic characteristics, exposure, mental health history, previous traumatic or difficult events, training and social isolation, COVID-19 concern by first responder category (wave 2 ESPA 13 November survey), n=428.

	Total		Health professionals		Affiliated volunteers		Firefighters		Police officers		p
	n	(%)	n	(%)	n	(%)	n	(%)	n	(%)	
**Gender**											**<0.0001**
Female	123	(28.7)	66	(61.1)	24	(32.9)	14	(7.4)	19	(32.2)	
Male	305	(71.3)	42	(38.9)	49	(67.1)	174	(92.6)	40	(67.8)	
**Mean age (SD)** (10 MV[#])	41.1 (10 .2)		48.6 (11.0)		38.4 (8.8)		36.5 (7.6)		45.3 (8.1)		**<0.0001**
**Level of study** (1 MV)											**<0.0001**
No high-school degree	45	(10.5)	3	(2.8)	7	(9.6)	34	(18.2)	1	(1.7)	
High-school diploma	101	(23.6)	4	(3.7)	9	(12.3)	74	(39.6)	14	(23.7)	
Graduate or post-graduate diploma	281	(65.8)	101	(93.5)	57	(78.1)	79	(42.2)	44	(75.6)	
**Intervention category (exposure)**											**<0.0001**
1- in unsecured crime scenes *	217	(50.7)	11	(10.2)	40	(54.8)	145	(77.1)	21	(35.6)	
2- in secured or remote crime scenes **	114	(26.6)	35	(32.4)	20	(27.4)	35	(18.6)	24	(40.7)	
3- only during the 3 weeks following the attacks	97	(22.7)	62	(57.4)	13	(27.4)	8	(4.3)	14	(23.7)	
**History of antidepressant use** (37 MV)	20	(5.1)	8	(8.1)	5	(7.3)	5	(3.0)	2	(3.6)	0.21
**History of mental health follow-up** (38 MV)	45	(11.5)	16	(16.2)	10	(14.5)	11	(6.6)	8	(14.8)	0.06
**Living alone** (4 MV)	78	(18.4)	26	(24.3)	23	(31.5)	18	(9.7)	11	(18.64)	**0.0002**
**Number of somatic problems present**	1.73 (2.0)		2.04 (2.0)		1.86 (1.9)		1.27 (1.8)		2.48 (2.0)		**<0.0001**

	Total		Health professionals		Affiliated volunteers		Firefighters		Police officers		p
	**n**	**(%)**	**n**	**(%)**	**n**	**(%)**	**n**	**(%)**	**n**	**(%)**	
**after the attacks of 13 November 2015 (SD)** (16 MV)											
**Traumatic events prior to the attacks** (64 MV)											0.11
No	197	(54.1)	46	(50.5)	36	(59.0)	83	(51.9)	32	(61.5)	
Intentional traumatic event	57	(15.7)	18	(19.8)	11	(18.0)	18	(31.6)	10	(19.2)	
Unintentional traumatic event	110	(30.2)	27	(29.7)	14	(22.9)	59	(36.9)	10	(19.2)	
**Traumatic events after to the attacks** (60 MV)	225	(61.1)	54	(56.3)	32	(52.5)	101	(63.1)	38	(74.5)	0.07
**Intervention in other terrorist events** (36 MV)											**0.0003**
Yes and listed this as a difficult situation	9	(2.3)	3	(3.0)	0	(0.0)	5	(3.0)	1	(1.8)	
Yes but did not list this as a difficult situation	161	(41.1)	48	(48.0)	31	(44.9)	48	(28.6)	34	(61.8)	
No	222	(56.6)	49	(49.0)	38	(55.1)	115	(68.4)	20	(36.4)	
**Psychological first aid training** (36 MV)											**<0.0001**
After or before	85	(21.7)	15	(15.0)	23	(33.8)	41	(24.3)	6	(10.9)	
Before and after	144	(36.7)	49	(49.0)	30	(44.1)	57	(33.7)	8	(14.6)	
Neither before nor after	163	(41.6)	36	(36.0)	15	(22.1)	71	(42.0)	41	(74.5)	
**Awareness of the psychological consequences that can be experienced after this type of traumatic intervention** (36 MV)											**<0.0001**

	Total		Health professionals		Affiliated volunteers		Firefighters		Police officers		p
	**n**	**(%)**	**n**	**(%)**	**n**	**(%)**	**n**	**(%)**	**n**	**(%)**	
After or before	117	(29.8)	23	(23.0)	13	(18.4)	61	(36.3)	20	(36.4)	
Before and after	214	(54.6)	67	(67.0)	47	(68.1)	83	(49.4)	17	(30.9)	
Neither before nor after	61	(15.6)	10	(10.0)	9	(14.7)	24	(24.3)	18	(29.5)	
**Low moral support** (41 MV)	14	(3.6)	2	(2.0)	1	(1.5)	6	(3.6)	5	(9.1)	0.10
**Low financial support** (40 MV)	93	(24.0)	32	(32.3)	10	(14.9)	34	(20.4)	17	(30.9)	**0.02**
**Low everyday social support** (40 MV)	57	(14.7)	18	(18.2)	9	(13.4)	24	(14.3)	6	(10.9)	0.64
**Social isolation** (41 MV)	53	(13.7)	13	(13.7)	11	(16.4)	19	(11.4)	10	(18.2)	0.55
**Knowing someone who could help regarding psychosocial risks** (36 MV)	281	(71.7)	81	(81.0)	51	(73.9)	113	(67.3)	36	(65.5)	0.07
**Relatives directly threatened by terrorists and/or physically or psychologically injured during the attacks** (1 MV)	60	(14.0)	11	(10.3)	12	(16.4)	30	(16.0)	7	(11.9)	0.49
**Concern about the COVID-19 epidemic (scale of 0 to 10)**											**0.002**
**0-3**	194	(45.3)	35	(32.4)	30	(41.1)	102	(54.3)	27	(45.8)	
**3-7**	173	(40.4)	49	(45.4)	28	(38.4)	71	(37.8)	25	(42.4)	
**7-10**	61	(14.2)	24	(22.2)	15	(20.5)	15	(8.0)	7	(11.9)	
**Total**	428	(100.0)	108	(25.2)	73	(17.1)	188	(43.9)	59	(13.8)	

Missing Value.
*for people who both intervened in unsecured crime scenes (1) and intervened during the 3 following weeks (3), exposure category (1) is retained.
** for people who both intervened in secured crime scenes or were distant from the crime scene during the night of the attack (2) and intervened during the 3 following weeks (3), exposure category (2) is retained.

Table S2: Multivariate regression analysis of the comparison between those who participated only in wave 1 and those who participated in both waves.

	OR	CI	p
**PTSD**			0.05
No PTSD	1.00	-	
Partial PTSD	0.77	0.49-1.22	0.26
PTSD	**0.41**	**0.19-0.88**	**0.02**
**Gender**			
Male	1.00	-	
Female	1.04	0.7 0-1.53	0.86
**First responder category**			0.05
Firefighters	1.00	-	
Affiliated volunteers	1.48	0.94-2.42	0.11
Police officers	0.96	0.56-1.65	0.89
Health professionals	**1.88**	**1.08-3.26**	**0.02**
**Intervention category (exposure)**			0.39
1- On the evening of November 13 and at unsecured crime scenes*	0.74	0.48-1.14	0.17
2- The evening of November 13 and at secured crime scenes or at a distance from the crime scenes**	1.00	-	
3- Only during the 3 weeks following the attacks	0.92	0.57-1.48	0.73
**Low everyday social support**			
No	1.00	-	
Yes	**0.53**	**0.3 0-0.93**	**0.03**

*for those who responded to both unsecured crime scenes (1) and those who responded within the following 3 weeks (3), exposure category (1) is retained.

** For persons who responded to both secured crime scenes or who were away from the crime scenes on the night of the attack (2) and who responded within the following 3 weeks (3), exposure category (2) is retained.
